# Intra-erythrocytes magnesium deficiency could reflect cognitive impairment status due to vascular disease: a pilot study

**DOI:** 10.1186/s12967-020-02645-w

**Published:** 2020-12-03

**Authors:** Clementina Sitzia, Michele Sterlicchio, Calogero Crapanzano, Elena Dozio, Elena Vianello, Massimiliano Marco Corsi Romanelli

**Affiliations:** 1 Department of Biomedical Sciences for Health, University of Milan, Via Luigi Mangiagalli, 31 -20133 Milan, Italy; 2 Neurophysiology Unit, IRCCS Policlinico San Donato, San Donato Milanese, Milan, Italy; 3 O.U. Clinical Pathology, Istituto Ortopedico Gaetano Pini, Milan, Italy; 4 O.U. Service of Laboratory Medicine 1-Clinical Pathology, Department of Pathology and Laboratory Medicine, I.R.C.C.S. Policlinico San Donato, San Donato Milanese, Milan, Italy

**Keywords:** Magnesium deficiency, Intra-erythrocytary measurement, Vascular cognitive impairment

## Abstract

**Background and aims:**

Magnesium is a fundamental cation that regulates neuronal transmission, protein synthesis, energy metabolism. Magnesium deficiency mostly affects nervous and cardiovascular systems determining weakness, tremors, seizure and arrhythmias. This condition retains also a role in memory function and neuronal plasticity. Importantly magnesium deficiency could remain latent and asymptomatic resulting a risk factor for cardiovascular disease. In this sense we aim to determine magnesium status in patient presenting cognitive impairment of vascular origin.

**Methods:**

21 healthy subjects and 27 patients presenting vascular cognitive impairment were included in this study. Both plasma and intraerythrocitary magnesium level were measured to detect magnesium deficiency and cognitive performance was evaluated trough Mini Mental State Evaluation (MMSE).

**Results:**

Here we showed that patients presenting vascular cognitive impairment present intraerythrocitary magnesium level lower than age-matched healthy subjects. To note their plasma magnesium resulted within reference limit.

**Conclusion:**

We suggest that intracellular magnesium laboratory measurement is needed to detect occult magnesium deficiency in population at risk. Magnesium supplementation could represent an adjuvant for healthy aging in high risk population.

## Background

Magnesium is a fundamental cation involved as a cofactor in many different enzymatic reactions that regulate energy metabolism and protein synthesis [1]. Magnesium is the second most abundant intracellular cation suggesting that its deficiency could impair different apparatus. In particular, symptoms of magnesium deficiency involve mostly neuromuscular and central nervous systems determining weakness, tremors, depression, psychosis and seizure. Other manifestations include ECG alteration, arrhythmias and metabolism disorders such as hypokalaemia and hypocalcaemia [1]. However, chronic hypomagnesaemia could result asymptomatic or cause aspecific symptoms so that laboratory testing represents a fundamental tool to reach the diagnosis. Hypomagnesaemia is considered to be a risk factor in a multitude of pathologies like atherosclerosis, coronary heart disease and diabetes mellitus [2, 3]. Magnesium is also correlated to mortality in cardiovascular patients with an unknown mechanism [4]. Indeed, occult hypomagnesemia has been reported in aged people and in neurodegenerative disorder such as Alzheimer Disease (AD) [5]. In this pilot study, we compared plasma and intra-erythrocytes magnesium level in a cohort of patients presenting cognitive impairment due to cerebrovascular disease to highlight the importance of magnesium measurement and supplementation in targeted populations.

## Materials and methods

### Study population

A total of 21 healthy subjects (mean age: 67.9 ± 5.1 years) and 27 patients (mean age: 71.8 ± 8,7 years) were included in this study: all the individuals without regard to the sex were over 60 years-old. Patients presenting vascular cognitive impairment were included in the study at the Neurophysiopathology Unit of Policlinico San Donato, San Donato Milanese (MI), Italy. Exclusion criteria were magnesium containing drug usage, renal insufficiency with a filtration rate < 60 ml/min, acute illness. Cognitive impairment was evaluated through Mini Mental State evaluation (MMSE), a widely used brief cognitive assessment tool [6, 7]. MMSE < 18 was considered as severe cognitive impairment, MMSE between 18 and 24 was considered mild impairment and MMSE > 24 was considered as normal test result. Neurological examination was performed in the Neurophysiopathology Unit of Policlinico San Donato, San Donato Milanese (MI), Italy. Healthy participants were recruited in Neurophysiopathology and Cardiovascular Unit of Policlinico San Donato during their clinical examination. Patients that performed magnesium screening for clinical reasons and evaluated for MMSE were included and, among them, those that had a score > 24 were considered to not present cognitive impairment. Exclusion criteria were also: magnesium containing drug usage, cancer or chronic disease, infections, severe reduction of kidney or liver function, diagnosis of dementia of neurodegenerative origin (Parkinson Disease, Alzheimer Disease, Multiple Sclerosis) and sign or suspect of cerebrovascular disease. Each patient gave the written informed consent for the research. This study was authorized by the Institutional Ethics Committee (ASL MI2-Melegnano via VIII Giugno, Milan) and was conducted according to the principles expressed in the Declaration of Helsinki (2008), the European Directive 2001/20/EC, the institutional regulation and Italian laws and guidelines.

### Sample collection and magnesium measurement

Regarding intra-erythrocyte magnesium measurements, 5 ml blood in one heparinized tube and one EDTA tube was collected from each patient after a fasting period of at least 8 h. 100μL of whole blood were added to 2 ml of 2% ascorbic acid in pure water in order to obtain lysed erythrocyte cells. Heparinized tube was centrifuged in order to separate plasma. Magnesium levels in the present lysed cells and in previously obtained plasma were measured on Beckman Coulter AU480 Chemistry Analyser (Brea, CA). EDTA tube was used to measure Haematocrit in automated haematology analyser XE-2100 (Sysmex). Results were calculated as follows: intra-erythrocytes Mg = [lysed cells Mg*21 (dilution factor)*Haematocrit (HCT)]/100 and expressed as mg/dL RBC (Red Blood Cells). Reference values: 1,98–3,21 mmol/L (4,56–7,8 mg/dL) as described in [8].

### Statistical analysis

Statistical analysis was performed using Prism GraphPad Software (San Diego, CA). T test analysis was applied between two groups to compare mean, assuming 95% confidence level. Two-sided values of p < 0.05 were considered as statistically significant. Correlation of Spearman and linear regression analysis were performed to evaluate correlation between plasma and erythrocytes magnesium level and magnesium deficiency and cognitive impairment.

## Result

**Fig. 1:**
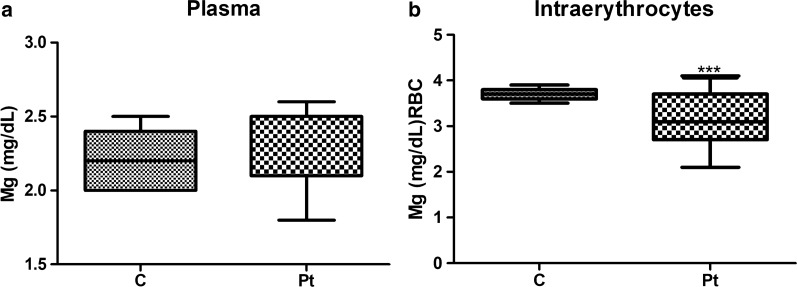
Magnesium measurement Plasma (**a**) and intraerytrocitary (**b**) Magnesium level in healthy subjects (**c**) and in patients (Pt) presenting cognitive impairment (p < 0001)

Patients with vascular cognitive impairment and age-matched healthy subjects were included in this study. Clinical Features of included patients are shown in Table 1. Patients group included both stroke and non-stroke patients, most of them presented major vascular risk factors such as hyperytension or atrial fibrillation. Mean Magnesium plasma level was 2243 ± 0189 in healthy subjects’ group (n = 21) and 2215 ± 0240 in Patients group (n = 27) (Fig. 1a). Both patients and healthy subjects had plasma magnesium level within reference values (Plasma RV: 1,58–2,55 mg/dl) and we did not observe any significant difference in magnesium level. However intracellular magnesium measurement revealed magnesium deficiency in patients with cognitive impairment suggesting a correlation between magnesium deficiency and the presence of cognitive impairment. Indeed mean intracellular Mg level was 3695 ± 0116 in healthy subjects’ group, whereas it was 3144 ± 0563 in patients’ group (p < 0.0001) (Fig. 1b). Correlation analysis demonstrated a positive relation between plasma and intra-erythrocytes magnesium measurement (Spearman r = 0,5163, p = 0.0058), however plasma measurement did not detect magnesium deficiency. We did not find any correlation between sex or age and magnesium measurement.

**Table 1:** Clinical features of included patients

Age	71,81 ± 8,79 Years (60-81)
Sex	45% M; 55% F
Stroke-free	37%
Stroke	72%
Hypertension	55%
Arrythmia/Atrial Fibrillation	27.27%
MMSE	18–25(29,61%)
	<18 (70,37%)

**Fig. 2:**
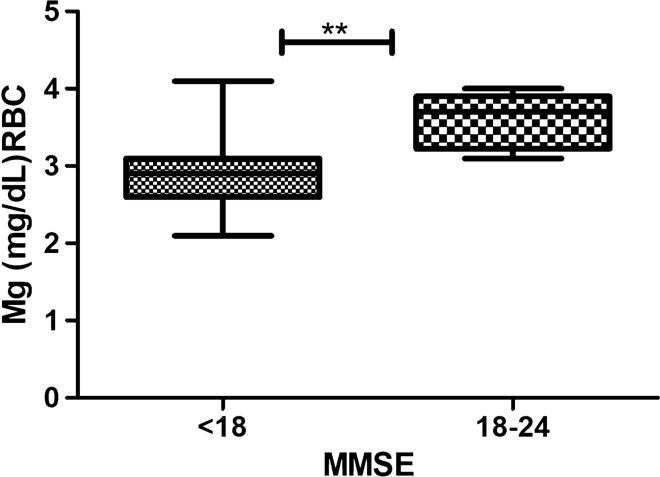
Magnesium measurement and disease severity Intraerytrocitary Magnesium level in patients with mild or severe cognitive impairment (p < 0.005)

Patients stratification, according to cognitive impairment grade, showed that patients with severe cognitive impairment (MMSE < 18) had reduced intraerythrocytary Mg level than patients with mild cognitive impairment (MMSE > 18 and < 24) (Mean RBC Mg 2.95 ± 0.12 and 3.59 ± 0.12 respectively; p < 0.005). (Fig. 2).

## Discussion

Magnesium deficiency has been recently considered to be a risk factor in different pathologies such as coronary heart disease, osteoporosis and hypertension. It has been demonstrated that prevalence of magnesium deficiency increased with age and that it is associated whit insulin resistance and diabetes mellitus. Furthermore, different studies linked magnesium deficiency to neurological disorders like Alzheimer Disease, memory disturbance and Major Depression. In this sense, concentration of magnesium affects N-methyl D-aspartate (NMDA) GABAA receptors function, influencing neuronal density and plasticity [9]. Here we showed that patients with vascular cognitive impairment presented intraerytrocitary magnesium deficiency despite maintaining normal magnesium plasma level. We showed that patients with cognitive impairment presented mean intra-erythrocytes Mg values significantly lower than healthy subjects and those with severe decline of brain function had even lower magnesium content. Nevertheless these findings are in line with recent literature linking magnesium deficiency with memory function and dementia [9]; further experiments in rat models demonstrated that Mg^++^ supplementation could compensate memory decline [10]. Similarly, antidepressant drugs restore intracellular magnesium level reducing intensity of symptoms in major Depression [11]. A possible explanation involves regulation of NMDA type receptors: interestingly Memantine (a drug that showed partial benefit both in Alzheimer disease and vascular dementia) blocks excessive NMDAR activity through a specific Mg- binding site in the NMDA channel [12].

Vascular cognitive impairment includes all degree of cognitive impairment from mild to severe up to dementia, in association to cerebrovascular disorder. This group includes both stroke and stroke-free patients independently from specific mechanism of vascular brain injury.

Vascular disease is the second cause of dementia, following Alzheimer Disease, but both forms could be present determining mixed cognitive impairment [13]. Although Magnesium level was also linked to vascular disease and cardiovascular mortality, the mechanism was not completely understood [4]. Brain injury could be caused by micro-infarcts, micro-haemorrhages and embolism, presenting the same risk factors than stroke and cardiovascular disease. In this sense, magnesium deficiency could determine ECG abnormalities and arrhythmia increasing the risk of Atrial Fibrillation and stroke and consequently dementia [14]. Indeed, hypomagnesaemia affects endothelial function at multiple levels favouring a pro-atherogenic and pro-thrombotic inflammatory environment that ultimately ends in plaque formation and progression, till plaque erosion or lumen blockade thus determining micro or macro infarct. In fact, hypomagnesaemia modulates HMG-CoA reductase, decreasing HDLs (high-density lipoproteins) and increasing total serum cholesterol, LDLs and triglycerides. Indeed, by favouring radical oxidant formation, it increases LDLs oxidation, a promoter event in plaque formation. In this sense it was demonstrated that high magnesium could prevent calcification of plaques [15] and, more importantly, in line with our results, there is a suggestive evidence that high Magnesium could prevent the risk of stroke [16]. In fact magnesium seems to be protective in animal model of white matter ischemia and glutamate-mediated necrosis [17, 18].

Indeed Mg deficiency promotes increased reactive-oxygen species (ROS) production and vasoconstriction in endothelial cells affecting vessels structure and function [19]; moreover, Mg2^++^ reduces platelet aggregation further preventing blood clotting. In last analysis, magnesium deficiency has been related to classical vascular risk factors as metabolic syndrome, diabetes (as it modulates insulin action promoting insulin resistance [20]) and atherosclerosis, suggesting that healthy diet and mineral supplementation are promising strategies to ameliorate cognitive performance later in life [2, 3]. Serum magnesium concentrations is reported to be significantly lower in diabetic patients than in normal subjects, probably favouring the progression on microangiopathy. Furthermore, reduced erythrocyte and platelet magnesium content was observed in patients with insulin-dependent diabetes mellitus, presenting microalbuminuria [21]. Our patient group was mixed including patients that presented stroke and micro-infarct or classicals cerebrovascular risk factor such as atrial fibrillation and hypertension,

Here, we demonstrated that plasma values, although correlated, did not reflect intracellular magnesium deficiency. This finding is probably due to chronic Mg deficiency that depleted intracellular store in favour of maintaining circulating plasma level, which is clearer in long-lasted red cells. These results are in line with extensive literature reporting intracellular measurement as a more reliable indicator of a long-term deficiency [22]. Moreover, alteration of erythrocytes membrane Mg^2+^/Na^+^ exchanger, leading to reduced intraerytrocytary Mg content, have been reported in a group of patient with Essential Hypertension [23]. In these patients Mg content negatively correlated with blood pressure. This finding suggested that multiple mechanism could be involved in intracellular Mg balancing. However, comparison between studies and conclusion are difficult to obtain because of variety of methods used and differences in units employed [22].

In this sense, the necessity of an easy approach to perform automated analysis to measure magnesium body level is emerging to overcome the diagnostic problems of “occult Mg” deficiency. These patients did not present specific symptoms or typical manifestations of magnesium deficiency, so that routinely screening could be reasonably proposed in patients at risk, especially because some studies demonstrated the reversibility of some clinical manifestation (such as memory and depression) after magnesium diet supplementation. Although presenting limitation due to limited number of patients, this study encourages the utilisation of intra-erythrocytes magnesium measurement for routinely screening program in patients with cognitive impairment of vascular origin. Further studies are needed to demonstrate whether magnesium deficiency is a consequence of inadequate dietary intake, a result of comorbidities in patients with cognitive impairment or a primary pathogenic factor. Little is known about prevalence of magnesium deficiency, ranging from 14.5% in general population to 35%–50% in older adult patients depending on study and compartment considered (plasma or intracellular measurement) [24, 25] till 60% of subjects with alcohol dependence (Alcohol withdrawal syndrome) [26]. In this sense, elderly people tend to reduce food and green vegetables intake—which could account for magnesium deficiency—however comorbidities such as gastrointestinal dysfunction and disease could represent an important cause of magnesium malabsorption. We did not observe a correlation between age and Mg content, however our study included subjects from 60 to 80 years to focus on vascular impairment and sample size was too small to elucidate this point.

## Conclusion

In conclusion, we observed reduced magnesium content in patients presenting vascular dementia. Since endothelium dysfunctions associated to low magnesium medium, are reversible following magnesium supplementation (such as normal proliferation rate, increase of NO release and reduction of MMP9 and NFKB activity [27]), strategies aimed to screen and increase intracellular magnesium content are fundamental to counteract vascular dysfunction due to physiological aging process, diabetes or unhealthy life styles promoting obesity and atherosclerosis. This property coupled to a neuroprotective effect of magnesium represents a powerful and easy tool to preserve and maintain brain function during late adulthood.

## Data Availability

The datasets used and/or analysed during the current study are available from the corresponding author on reasonable request.

## References

[1] Elin RJ (1994). Magnesium: the fifth but forgotten electrolyte. Am J Clin Pathol.

[2] Liao F, Folsom AR, Brancati FL (1998). Is low magnesium concentration a risk factor for coronary heart disease? The Atherosclerosis Risk in Communities (ARIC) Study. Am Heart J.

[3] Lopez-Ridaura R, Willett WC, Rimm EB, Liu S, Stampfer MJ, Manson JE (2004). Magnesium intake and risk of type 2 diabetes in men and women. Diabetes Care.

[4] Kieboom BC, Niemeijer MN, Leening MJ, van den Berg ME, Franco OH, Deckers JW, et al. Serum Magnesium and the Risk of Death From Coronary Heart Disease and Sudden Cardiac Death. Journal of the American Heart Association. 2016;22;5(1). 10.1161/JAHA.115.002707 PMC4859391 26802105

[5] Vural H, Demirin H, Kara Y, Eren I, Delibas N (2010). Alterations of plasma magnesium, copper, zinc, iron and selenium concentrations and some related erythrocyte antioxidant enzyme activities in patients with Alzheimer’s disease. J Trace Elem Med Biol.

[6] Folstein MF, Folstein SE, McHugh PR. “Mini-mental state”. A practical method for grading the cognitive state of patients for the clinician. Journal of psychiatric research. 1975;12(3):189-98. 10.1016/0022-3956(75)90026-6 1202204

[7] Trivedi D (2017). Cochrane Review Summary: mini-Mental State Examination (MMSE) for the detection of dementia in clinically unevaluated people aged 65 and over in community and primary care populations. Primary Health Care Res Develop.

[8] Abbott A (2003). Cell culture: biology’s new dimension. Nature.

[9] Moykkynen T, Uusi-Oukari M, Heikkila J, Lovinger DM, Luddens H, Korpi ER (2001). Magnesium potentiation of the function of native and recombinant GABA(A) receptors. NeuroReport.

[10] Xiong W, Liang Y, Li X, Liu G, Wang Z. Erythrocyte intracellular Mg(2 +) concentration as an index of recognition and memory. Scientific reports. 2016; 2;6:26975. 10.1038/srep26975 PMC4890594 27253451

[11] Nechifor M (2009). Magnesium in major depression. Magnes Res.

[12] Chen HS, Lipton SA (2005). Pharmacological implications of two distinct mechanisms of interaction of memantine with N-methyl-D-aspartate-gated channels. J Pharmacol Exp Ther.

[13] Dichgans M, Leys D (2017). Vascular Cognitive Impairment. Circ Res.

[14] Diener HC, Hart RG, Koudstaal PJ, Lane DA, Lip GYH (2019). Atrial fibrillation and cognitive function: jACC review topic of the week. J Am Coll Cardiol.

[15] Hruby A, O’Donnell CJ, Jacques PF, Meigs JB, Hoffmann U, McKeown NM. Magnesium intake is inversely associated with coronary artery calcification: the Framingham Heart Study. JACC Cardiovascular imaging. 2014;7(1):59-69. 10.1016/j.jcmg.2013.10.006 PMC3957229 24290571

[16] Veronese N, Demurtas J, Pesolillo G, Celotto S, Barnini T, Calusi G, et al. Magnesium and health outcomes: an umbrella review of systematic reviews and meta-analyses of observational and intervention studies. European journal of nutrition. 2019; 25. 10.1007/s00394-019-01905-w 30684032

[17] Finkbeiner S, Stevens CF. Applications of quantitative measurements for assessing glutamate neurotoxicity. Proceedings of the National Academy of Sciences of the United States of America. 1988;85(11):4071-4. 10.1073/pnas.85.11.4071 PMC280363 2453886

[18] Stys PK, Ransom BR, Waxman SG (1990). Effects of polyvalent cations and dihydropyridine calcium channel blockers on recovery of CNS white matter from anoxia. Neurosci Lett.

[19] Zhou Q, Olinescu RM, Kummerow FA (1999). Influence of low magnesium concentrations in the medium on the antioxidant system in cultured human arterial endothelial cells. Magnes Res.

[20] Barbagallo M, Dominguez LJ (2007). Magnesium metabolism in type 2 diabetes mellitus, metabolic syndrome and insulin resistance. Arch Biochem Biophys.

[21] Allegra A, Corsonello A, Buemi M, D’Angelo R, di Benedetto A, Bonanzinga S (1997). Plasma, erythrocyte and platelet magnesium levels in type 1 diabetic patients with microalbuminuria and clinical proteinuria. J Trace Elem Med Biol.

[22] Walther LE, Winnefeld K, Solch O (2000). Determination of iron, copper, zinc, magnesium and selenium in plasma and erythrocytes in neurosurgical patients. J Trace Elem Med Biol.

[23] Picado MJ, de la Sierra A, Aguilera MT, Coca A, Urbano-Marquez A (1994). Increased activity of the Mg2 +/Na + exchanger in red blood cells from essential hypertensive patients. Hypertension.

[24] Schimatschek HF, Rempis R (2001). Prevalence of hypomagnesemia in an unselected German population of 16,000 individuals. Magnes Res.

[25] Ulger Z, Halil M, Kalan I, Yavuz BB, Cankurtaran M, Gungor E (2010). Comprehensive assessment of malnutrition risk and related factors in a large group of community-dwelling older adults. Clin Nutr.

[26] Maguire D, Talwar D, Burns A, Catchpole A, Stefanowicz F, Robson G, et al. A prospective evaluation of thiamine and magnesium status in relation to clinicopathological characteristics and 1-year mortality in patients with alcohol withdrawal syndrome. Journal of translational medicine. 2019;17(1):384. 10.1186/s12967-019-02141-w PMC6873772 31752901

[27] Maier JA (2012). Endothelial cells and magnesium: implications in atherosclerosis. Clin Sci.

